# Protective Effect of Mesenchymal Stem Cell-Derived Extracellular Vesicles on Inner Ear Sensorineural Cells Affected by Cisplatin

**DOI:** 10.3390/medicina61061042

**Published:** 2025-06-05

**Authors:** Maria Perde-Schrepler, Ioana Brie, Mihai Cenariu, Sergiu Chira, Lajos Raduly, Liviuta Budisan, Ioana Berindan-Neagoe, Rares Stiufiuc, Maximilian Dindelegan, Cristina Blebea, Emoke Pall, Alma Aurelia Maniu

**Affiliations:** 1 Department of Otoryhinolaryngology, Iuliu Hatieganu University of Medicine and Pharmacy, 400012 Cluj-Napoca, Romania; blebea.cristina@elearn.umfcluj.ro (C.B.); aurelia.maniu@umfcluj.ro (A.A.M.); 2 Radiobiology and Tumor Biology Department, Ion Chiricuta Institute of Oncology, 400015 Cluj-Napoca, Romania; brie.ioana@iocn.ro (I.B.); maximilian.dindelegan@umfcluj.ro (M.D.); 3 Department of Clinical Sciences, University of Agricultural Sciences and Veterinary Medicine of Cluj-Napoca, 400372 Cluj-Napoca, Romania; 4 Genomics Research Centre, Iuliu Hatieganu University of Medicine and Pharmacy, 400012 Cluj-Napoca, Romanialajos.raduly@umfcluj.ro (L.R.); liviuta.petrisor@umfcluj.ro (L.B.);; 5 Bio-Nano-Physiscs Department, Iuliu Hatieganu University of Medicine and Pharmacy, 400012 Cluj-Napoca, Romania; 6 Department of Surgery- Practical abilities, Iuliu Hatieganu University of Medicine and Pharmacy, 400012 Cluj-Napoca, Romania

**Keywords:** extracellular vesicles, mesenchymal stem cells, sensorineural inner ear cells

## Abstract

*Background and Objectives*: Extracellular vesicles (EVs) derived from mesenchymal stem cells have gained much attention as potential therapeutic agents in many diseases, including hearing disorders such as sensorineural hearing loss (SNHL). EVs inherit similar therapeutic effects, including the stimulation of tissue regeneration from the parental cells. The aim of our study was to isolate EVs produced by MSCs and use them to treat inner ear cells in culture to evaluate their protective potential against the damaging effect of an ototoxic drug. *Materials and Methods*: We isolated MSC-derived EVs by precipitation and characterized them by number, size, and morphology using nanoparticle tracking analysis and TEM, evaluated the protein concentration by BCA assay and the presence of EV markers CD9, CD63, and CD81 by the Dot Blot immunoblotting method. HEI-OC1 inner ear cell line was treated with EVs either alone or followed by Cisplatin. We assessed the uptake of EVs in HEI-OC1 cells by fluorescence microscopy after PKH26 labeling, ROS production by the DCFDA (dichlorfluorescein diacetate) assay, cellular viability by Alamar Blue assay, and apoptosis with the Annexin V/Propidium Iodide method. *Results*: The isolated EVs had mean dimensions of 184.4 nms and the concentration of the EV suspension was 180 × 10^6^ particles/mL. TEM analysis showed intact vesicular structures with lipid-bilayer membranes having similar sizes with those measured by NTA. The PKH26-labeled EVs were observed in the HEI-OC1 cells after 24 h incubation, the amount increasing with the concentration. EVs reduced ROS production and increased the number of viable cells both alone and as pretreatment before Cisplatin, dose-dependently. Cells in early apoptosis were inhibited by EVs, while those in late apoptosis were enhanced, both with and without Cisplatin. *Conclusions*: EVs secreted by MSC protected HEI-OC1 cells against Cisplatin toxicity, reduced ROS production, and stimulated cell viability and the elimination of damaged cells by apoptosis, protecting the HEI-OC1 cells against Cisplatin-induced damage.

## 1. Introduction

According to the World Health Organization, about 5% of the world’s population suffer from disabling hearing loss [1,2]. In over 90% of the cases, hearing loss occurs due to the damage of inner ear cells or the auditory nerve: sensorineural hearing loss (SNHL) caused by exposure to extreme noise, ototoxic drugs, aging, etc. [3].

As treatment options are scarce and mostly ineffective [4], including hearing aids and cochlear implants which cannot adequately restore normal hearing [5], new alternatives are studied, such as biological therapies based on stem cell technology. Stem cell therapies are gaining more and more attention due to their substantial regenerative potential. These include cellular therapies using mesenchymal stem cells (MSCs) as well as their products: extracellular vesicles (EVs).

MSCs are the most common adult or somatic stem cells. Among them, bone marrow-derived MSCs (BM-MSCs) not only have differentiation capacity for multiple lineages (osteocytes, chondrocytes, adipocytes) but also exhibit anti-inflammatory properties and augment tissue regeneration [6].

Extracellular vesicles (EVs) are cell-derived vesicles surrounded by a bilayered lipid membrane containing bioactive molecules that are characteristic for the cells of origin and which are delivered to recipient cells. Based on their biogenesis mechanism and size, EV are classified as exosomes (30–150 nm)—endosome-originated EVs generated in three steps: biogenesis, transport, and release; micro-vesicles (100–1000 nm)—formed by outward budding and shedding from the plasma membrane; and apoptotic bodies (>1000 nm)—generated in the process of apoptosis [7]. The International Society for Extracellular Vesicles recommends using the name small EV for EVs < 200 nm and for the ones > 200 nm, large EV [8,9].

The rationale for using stem cells or EVs to treat SNHL is based on the MSC capacity to induce tissue regeneration, to secrete neuroprotective growth factors, to downregulate excessive fibrosis and to modulate immune response as proved in several in vitro [10] and in vivo studies [11,12,13,14].

EVs have also been demonstrated to protect against drug-induced hearing loss by reducing the apoptosis of mouse cochlear hair cells [15].

To date, there are still no advanced clinical studies using MSCs or EVs for the inner ear pathology, the majority of clinical trials using MSCs for hearing loss being in phases I/II [16]. The use of EVs in regenerative medicine is an attractive alternative to cell therapy because of their important advantages: low immunogenicity, easy accessibility, and reduced ethical concerns.

The aim of our study was to evaluate the efficacy of mouse MSC-derived EVs in the protection of sensorineural cells against Cisplatin-induced ototoxicity.

## 2. Materials and Methods

### 2.1. Isolation of Mesenchymal Stem Cells from Mouse Bone Marrow and the Characterization of the MSC Culture

#### 2.1.1. Isolation

The animal experiments were approved by the ethical committee of Iuliu Hatieganu University of Medicine and Pharmacy and The National Sanitary Veterinary and Food Safety Authority, Cluj-Napoca. Four male CD1 mice aged 3–4 weeks were euthanized. The hind leg long bones (femur, tibia) were dissected and placed in a Petri dish containing DMEM supplemented with Penicillin-Streptomycin. The ends of the bones were cut and the needle of a 10 mL syringe containing growth medium was inserted in the bone, flushing the marrow plug. The obtained suspension was filtered through a 70 µm Filcons filter to remove debris, centrifuged at 1000 rpm for 5 min, and the cells were counted, then seeded on cell culture flasks at a cellular density of 2 × 10^6^ cells/cm^2^ in complete stem cell culture medium: DMEM/F12 (1:1 vol), 15% FBS, 1% sodium pyruvate, 1% β-mercaptoethanol, 1% NEA, 1% penicillin-streptomycin, and 1% Gluthamin. All the culture media were from Sigma Aldrich, Chemie GmbH, Taufkirchen, Germany. After 24 h, the non-attached cells were discarded, and the medium was changed. The medium was changed every 3–4 days and the cells were passaged when reaching confluence. The BM-MSC cultures were observed daily using a ZEISS Axio Observer D1 inverted fluorescence microscope with Axiovision SE 64 Rel 4.8. software.

#### 2.1.2. The Assessment of MSC Markers by Flow Cytometry and Fluorescence Microscopy

The presence of MSC markers and the absence of hematological markers was assessed by flow-cytometry using a FACS Canto II flow-cytometer with FACS Diva 6.1.3 software (Becton Dickinson, Franklin Lakes, NJ, USA). We used fluorescently conjugated antibodies: mouse anti-CD73 conjugated with AF750, anti-CD105 conjugated with FITC, anti-CD90 conjugated with PE, and anti-CD34. All antibodies were from R&D Systems, Minneapolis, MN, USA. The labeled cells were also observed by fluorescence microscopy. The samples were fixed in 4% paraformaldehyde (PFA) for 20 min and permeabilized with 0.5% Triton X-100 for 15 min. After washing with PBS, the samples were incubated with the above-mentioned antibodies. The cell nuclei were counter-stained with DAPI. The images were analyzed with a Nikon 600 Eclipse fluorescence microscope (Nikon Corporation, Tokyo, Japan) equipped with a DS-Ri2 color digital camera and analyzed using the NIS-Elements D image analysis software (Version 5.30.00 64 bit).

#### 2.1.3. The Differentiating Capacity of the Isolated MSCs

MSCs are multipotent stromal cells, characterized by their ability to differentiate in certain conditions into a variety of cell types such as adipocytes, osteoblasts, chondrocytes, etc.

BM-MSC differentiation was induced by culturing the cells in adipogenic, osteogenic, and chondrogenic differentiation media. The adipogenic differentiation medium contained complete stem cell medium supplemented with 0.1 µM dexamethasone, 1% BSA (bovine serum albumin), 1% ITS (insulin-transferrin-selenium), 1% triiodoyronin, and 0.1% Indomethacin. The cells were maintained in this medium for one week. After this interval, there were visible droplets of fat in the cells. For the visualization of the lipid droplets, staining with Oil red was performed after fixation with 4% PFA for 15 min and washing with isopropanol 60% for 1 min. The nuclei were counterstained with Giemsa.

For osteogenic differentiation, after BM-MSCs reached 80% confluence, the medium was changed with osteogenic differentiation medium consisting of complete stem cell culture medium supplemented with β-glycerophosphate 4%, ascorbic acid 35 µg/mL, and Dexamethasone 0.1 µM. After 3 weeks, the cells were fixed with 4% PFA, stained with Alizarin Red 2% for 45 min at pH 4.1–4.3, and then washed. Alizarin red stains the extracellular calcium deposits in mineralized BM-MSC-derived mature osteoblasts, which appear bright red.

The differentiation of BM-MSCs into chondrocytes was initiated in low-attached 6-well plates in chondrogenic medium: complete stem cell culture medium supplemented with Dexamethasone 0.1 µM, ascorbic acid 35 µg/mL, ITS 1%, and TGF β1-10 ng/mL. The cells formed conglomerates, and the medium was changed every 3–4 days carefully to not disturb the conglomerates. After 3 weeks of culture, the cell conglomerates were transferred to 4-well chamber slides (Thermo Fisher Scientific, Waltham, MA, USA), cultured for another week and then stained with Alcian Blue solution 3% in acetic acid, pH 2.5, for 30 min. Alcian blue stains both sulfated and carboxylated the acid mucopolysaccharides and glycoproteins characteristic for the chondrocytes extracellular matrix.

The differentiated cells were observed with a Nikon 600 Eclipse microscope and the images were recorded with a DS-Ri2 color digital camera and analyzed using the NIS-Elements D image analysis software (Version 5.30.00 64 bit, Nikon Corporation, Tokyo, Japan).

### 2.2. Isolation and Characterization of Extracellular Vesicles (EV)

When BM-MSCs cultured in a 75 cm^2^ culture flask reached 80% confluence, the medium was changed with FBS free medium for 24 h before exosome isolation. The supernatant was collected from the flasks and centrifuged at 2000× *g* for 30 min to remove cells or cell debris, then mixed with the exosome isolation reagent (Invitrogen) in a 2:1 volumetric proportion and kept overnight at 4 °C. The next day, the supernatant–reagent mix was centrifuged at 10,000× *g* for 60 min at 4 °C, and the obtained pellet was resuspended in 1 mL PBS. 

#### 2.2.1. The Protein Concentration

The protein concentration was evaluated using the micro-BCA kit (Thermo-Scientific, Waltham, MA, USA) according to the manufacturer’s protocol. The bicinchoninic acid (BCA) assay is based on the reduction of Cu^+2^ to Cu^+1^ in proteins in alkaline solution which leads to a color change (from green to purple). The absorbances of standard solutions (prepared with bovine serum albumin—BSA) and of the sample were read at 562 nm using a Spectramax 190 spectrophotometer (Molecular Devices, San Jose, CA, USA). The protein concentration of the EV solution was evaluated by comparing the measured absorbance of the sample with the standard curve.

#### 2.2.2. Evaluation of EV Markers Using the Dot Blot Immunobinding Assay and Western Blot

The Dot Blot technique involves the application of the sample directly to a membrane, where it is probed with a specific antibody. After adding a detection enzyme, the intensity of the signal is proportional to the amount of the target protein. A nitrocellulose membrane (Bio-Rad Laboratories, Hercules, CA, USA) was pre-wet in 1× Trys-buffered saline-TBS (Bio-Rad) and assembled in the Bio-Dot Microfiltration System (Bio-Rad). Then, 100 µL of 1xTBS was loaded in 6 wells and vacuum was applied until the wells were drained. Then, 300 µL of exosome sample was loaded in 3 wells, while other 3 wells were loaded with 1xTBS as negative controls. Samples and negative controls passed passively through the membrane, without vacuum. After drainage, the wells were washed by gravity flow with 300 µL of 1xTBS. Next, the wells were loaded with 200 µL of blocking solution (1% BSA in 1xTBS) and let to drain by gravity flow. After washing with 300 µL of TBST (0.05% Tween-20 in 1xTBS) and vacuum, the membrane was stained for 5 min in 0.5% Ponceau Red in 5% acetic acid for a rapid and reversible detection of protein blots. After the membranes were blocked for 1 h in 3% BSA in TBST, the primary antibodies were applied: rabbit monoclonal anti-CD9, dilution 1:500 in 3% BSA in TBST; mouse monoclonal anti-CD63, dilution 1:100 in 3% BSA in TBST; and rabbit monoclonal anti-CD81, dilution 1:000 in 3% BSA in TBST, and kept overnight at 4 °C (all primary antibodies from Novus Biological, Centennial, CO, USA). After washing 5 times in TBST, the secondary antibodies (Cell Signaling Technology, Danvers, Ma, USA) were added: anti-rabbit IgG, an HRP-linked antibody and anti-mouse IgG, and HRP-linked antibody; dilution 1:2500 in 3% BSA in TBST, for 1 h at room temperature. The imaging of the obtained blots was performed with Clarity Western ECL Substrate (Bio-Rad) and Azure Biosystem C300 Imager software (Azure^TM^ cSeries, Azure Biosystems, Dublin, CA, USA).

Western Blot. We used 3 different loading buffers: 10 µL 4× Laemmli Loading Buffer (without dithiotreitol -DTT); 10 µL 4× Laemmli Loading Buffer (50 mM DTT); and 10 µL 4× Laemmli Loading Buffer (50 mM DTT), added to 30 µL sample for 5 min at 95 °C. The mixture (3 × 40 µL) was loaded on TGX 4–20% Precast Gel. The transfer was achieved on PVDF membrane 0.2 µm using Trans-Blot Turbo RTA Transfer Kit. After blocking 1 h in 5% NFDM in TBST (0.1% Tween-20), the membrane was washed twice with TBST (0.05% Tween-20), for 5 min. The primary antibodies: rabbit monoclonal anti-CD9 (Novus NBP2-67310, MW antigen 25 kDa), dilution 1:1000 in 3% BSA in TBST (0.1% Tween-20); mouse monoclonal anti-CD63 (Novus NB100-77913, MW antigen 60 kDa), dilution 1:400 in 3% BSA in TBST (0.1% Tween-20); rabbit monoclonal anti-Annexin V [Cell Signaling #855, MW antigen 30 kDa, dilution 1:1000 in 5% BSA in TBST (0.1% Tween-20); rabbit monoclonal anti-caspase [Cell Signaling #14220, MW antigen 35 kDa (uncleaved), 17 and 19 kDa (cleaved)] dilution 1:1000 in 5% BSA in TBST (0.1% Tween-20); and rabbit monoclonal anti-Hsp70 (Cell Signaling, MW antigen 70 kDa), dilution 1:1000 in 5% BSA in TBST (0.1% Tween-20) were added and incubated overnight at 4 °C. After washing 5 times with TBST, for 10 min each, the secondary antibody was added: anti-rabbit IgG, an HRP-linked antibody (Cell signaling #7074), dilution 1:2500 in 3–5% BSA in TBST (0.1% Tween-20) or anti-mouse IgG, an HRP-linked antibody (Cell signaling #7076), dilution 1:2500 in 3–5% BSA in TBST (0.1% Tween-20), for 1 h at room temperature. After washing 5 times in TBST, Clarity Western ECL Substrate (Bio-Rad) was added. The images were taken on C300 Azure Biosystem chemidoc.

#### 2.2.3. The Size Distribution and Concentration of the Extracellular Vesicles

The size distribution and concentration of the extracellular vesicles were analyzed using nanoparticle tracking analysis (NTA) with Nanosight NS300 (Malvern Panalytica, Malvern, UK) and the corresponding software.

#### 2.2.4. TEM

The separated EVs were also analyzed using transmission electron microscopy. A total of 20 μL of the EV suspension was mixed with 20 μL of 2% ammonium molybdate solution with a pH around 6.5. Then, a glow-discharged, carbon-coated copper grid of 400 mesh was placed over the mixture drop for 30 s. The excess liquid was removed with filter paper, and the grid with the sample was dried for 10 min in a desiccator chamber. Thereafter, TEM images of the exosomes were taken on a Hitachi HT7700 electron microscope (Hitachi Ltd., Tokyo, Japan) equipped with an 8-megapixel CCD camera and operating at 80 kV in high-resolution mode.

### 2.3. Assessment of the Protective Effect of EVs on HEI-OC1 Cells Against Cisplatin Toxicity

#### 2.3.1. Culture of the HEI-OC1 Cells

The HEI-OC1 cell line, (House Ear Institute Los Angeles, CA, USA), was a gift from Prof. Federico Kalinek. They were cultured under permissive conditions: (33 °C, 10% CO_2_) in high glucose (4.5G) DMEM supplemented with 10% bovine fetal serum. (Sigma-Aldrich Chemie GmbH, Taufkirchen, Germany).

#### 2.3.2. Internalization of EVs into Hair Cells In Vitro

EVs (1.47 × 10^8^ particles in 500 µL) were fluorescently labeled with a PKH26 red fluorescent cell linker kit (Invitrogen). PKH26 is an amphiphilic dye which intercalates into bipolar membranes. EVs were incubated with 500 µL PKH26 0.2 µM for 5 min at room temperature. The reaction was stopped by adding 500 µL BSA 1%. The EVs were pelleted at 80,000× *g* for 2 h at 4 °C, washed with PBS, and centrifuged again in the same conditions. The EV pellet was resuspended in 500 µL PBS. HEI-OC1 cells were seeded on chamber slides (Nunc, Thermo Scientific, Waltham, MA, USA). Increasing dilutions of the labeled EV were added to the wells (50, 100, and 150 µL corresponding to 22.5, 45, and 67.5 µg proteins), in triplicate. Three wells were kept non-treated. After 24 h incubation, the cells were fixed with 4% paraformaldehyde (PFA) for 15 min, then washed twice with PBS. The slides were mounted with UltraCruz mounting medium (Santa Cruz Technologies, Dallas, TX, USA) and examined with Nikon 600 Eclipse fluorescence microscope (excitation 551 nm and emission 567 nm). The images were recorded with a DS-Ri2 color digital camera and analyzed using the NIS-Elements D image analysis software (Version 5.30.00 64 bit, Nikon Corporation, Tokyo, Japan).

#### 2.3.3. Evaluation of Reactive Oxygen Species (ROS) Production

DCFDA (dichlorfluorescein diacetate), (Thermo-Fisher Scientific, Waltham, MA, USA), a lipophilic and non-ionic fluorogenic probe capable of diffusing and crossing the cell membrane into the cytoplasm of the cells was used. It is deacetylated by intracellular esterases, producing the non-fluorescent compound 2′,7′–dichlorofluorescein (DCFH) which reacts with the intracellular ROS (hydrogen peroxide, peroxyl, and hydroxyl radicals) being converted to the highly fluorescent 2′,7′-dichlorofluorescein (DCF). HEI-OC1 cells were seeded on 96-well plates. The next day, 100 μL of DCFDA 10 μM was added to the wells in the dark. After 1 h incubation at 37 °C in a humidified 5% CO_2_, the wells were washed and the plates were treated with 100 μL culture medium for the non-treated control, 100 μL culture medium with 100 μM Cisplatin, 100 μL culture medium containing 3 dilutions of EV (EV1 = 10 µL/ 4.5 µg, EV2 = 20 µL/ 9 µg and EV3 = 30 µL/ 13.5 µg), and 100 μL culture medium with three dilutions of EVs followed after 30 min by 100 μM Cisplatin (the concentration of Cisplatin close to IC50, which was described elsewhere). All treatments were in triplicate. The emitted fluorescence readings were performed on a Biotek Synergy 2 microplate reader (BioTek Instruments, Winooski, VT, USA) at 15 min, 30 min, 1 h, 2 h, 4 h, and 24 h at 495 nm excitation and 530 nm emission. Wells with culture medium alone were considered background negative controls. Relative fluorescence units (RFU) were calculated by subtracting blank readings from all measurements.

#### 2.3.4. Evaluation of Cell Viability with Alamar Blue 

Alamar Blue is a cell viability assay reagent containing the fluorescent blue indicator dye called resazurin. This assay uses the natural reducing power of living cells to convert resazurin to fluorescent resorufin. The HEI-OC1 cells, seeded in 96-well plates at 15,000 well density, were treated with three concentrations of EV suspension (EV1, EV2, and EV3). After 30 min of incubation, Cisplatin 100 µM was added. Three wells were left untreated and three wells were treated only with Cisplatin, while the rest of the wells were treated either with EVs or with EVs followed by Cisplatin. After 4 h incubation, 10 µL Alamar Blue (in 100 µL medium) was added to each well. The emitted fluorescence was assessed on a BioTek 2 Synergy microplate reader (BioTek Instruments, Winooski, VT, USA) at 560 nm excitation and 590 nm emission.

#### 2.3.5. Evaluation of Apoptosis by the Annexin V/PI Flow-Cytometry Method

Apoptosis was assessed with the Annexin V-AlexaFluor 488/ PI Apoptosis Detection Kit (Thermo-Fisher Scientific, Waltham, MA, USA) according to the manufacturer’s instructions. Annexin V conjugates bind to the externalized phosphatidylserine in the early/intermediate stages of apoptosis. The cells were treated with EVs followed by Cisplatin, as described in the previous paragraph. After 4 h and 24 h incubation, the cells were collected by trypsinization, washed with PBS, and resuspended in 1X Binding Buffer at 1–5 × 10^6^ cells/mL. Then, 5 μL of fluorochrome-conjugated Annexin V and 1 μL of PI were added to 100 μL of the cell suspension and the cells were incubated for 15 min at room temperature in the dark. After adding 400 µL of binding buffer to each tube, the stained cells were analyzed on a FACS Canto II flow-cytometer, using FACS Diva version 6.1.3 software (Becton Dickinson, Franklin Lakes, NJ, USA). Four populations of differently labeled cells were assessed (percentages): normal cells (no labeling), early apoptotic cells (Annexin V+/PI−), late apoptotic cells (AnnexinV+/PI+), and necrotic cells (PI+).

### 2.4. Statistical Analysis

Statistical analysis was performed with the GraphPad Prism 5 software (San Diego, CA, USA) using the ANOVA analysis of variance to test the dose-dependence effect of the treatments followed by paired comparisons with Bonferroni and Dunnett post hoc tests for statistical significance. Statistical significance was considered for *p* < 0.05.

## 3. Results

### 3.1. BM-MSC Culture

**Figure 1:**
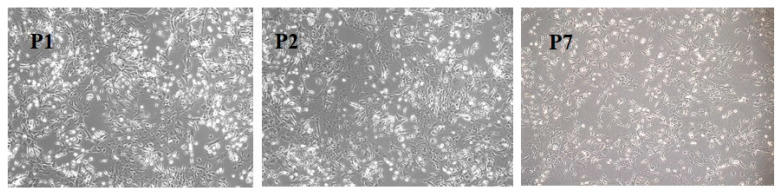
BM-MSCs in different passages (P1, P2, P7) observed under an inverted phase Zeiss microscope, magnification 100×.

The isolated BM-MSCs were observed daily and their morphology and growth rate were analyzed. After 7 passages, the morphology became uniform, with spindle-shaped cells (Figure 1). The cells were passaged every week. Reaching passage 17, when they were stored in liquid nitrogen, the growth rate was similar, indicating the preservation of the isolated cells’ proliferation capacity.

### 3.2. BM-MSC Characterization

#### 3.2.1. BM-MSC Differentiation

**Figure 2:**
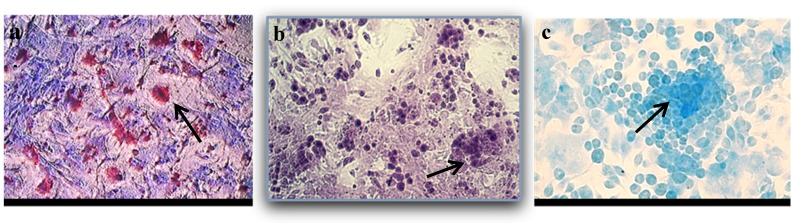
(**a**). Lipid droplets stained with Oil red (black arrow). (**b**). Extracellular calcium deposits produced by differentiated osteoblasts stained with Alizarin Red. (black arrow) (**c**). Alcian blue-stained mucopolysaccharides and glycoproteins characteristic for chondrocytes’ extracellular matrix (black arrow). (N = 3).

We tested the capability of the isolated cells to differentiate towards three lineages of connective tissues: bone, fat, and cartilage. After culture in specific differentiation inducing media, the BM-MSCs showed characteristic features of the tissues they differentiated into: lipid droplets stained in red with Oil red after culture in adipogenic medium for seven days; extracellular calcium deposits stained by Alizarin Red after 3 weeks in osteogenic medium, and the presence of mucopolysaccharides and glycoproteins characteristic for the chondrocytes extracellular matrix stained by Alcian Blue after three weeks in chondrogenic medium (Figure 2).

#### 3.2.2. The Presence of MSC Markers by Flow Cytometry and Fluorescence Microscopy

**Figure 3:**
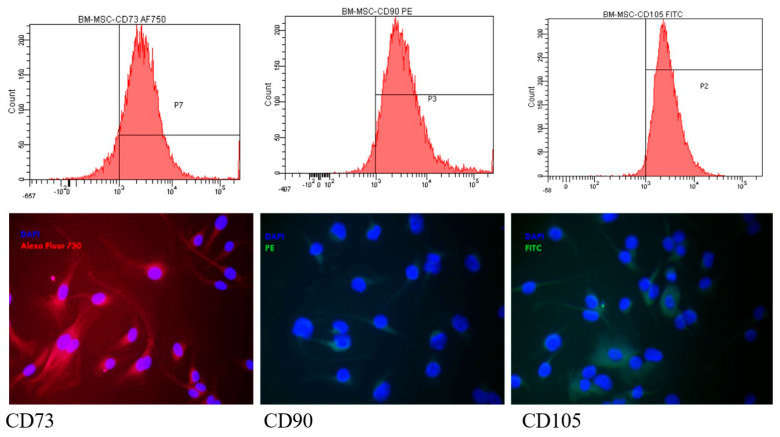
**Above row images**: Histograms representing the expression of mesenchymal stem cell-specific antigens: CD73, CD90, and CD105. On the x axis, the cells’ fluorescence is depicted (AF750, PE, FITC), while on the y axis, the number of the examined cells is represented. **Lower row**: Microscopic images of BM-MSCs fluorescently labeled with CD73 Alexa Fluor 750, CD90 labeled with PE, and CD105 FITC (nucleus counterstained with DAPI). Magnification 400×. (N = 3).

BM-MSCs evaluated by flow-cytometry at different passages showed the presence of characteristic mesenchymal cell markers: CD73 (>80.82 ± 6.45%), CD105 (>97.55 ± 1.9%), and CD90 (>91.28 ± 6.35%) (Figure 3—upper row) CD34, a hematological progenitor cell antigen, was absent for all passages (<0.02%). The presence of the markers was observed also with fluorescence microscopy: CD73—red fluorescence in the cytoplasm (AF750); CD105 was evidenced as green fluorescence (FITC), and CD90 appeared as red fluorescence (PE). The nuclei were visualized in blue with DAPI (Figure 3—lower row).

### 3.3. Isolation and Characterization of EVs

#### 3.3.1. EV Size, Concentration, and Morphology by Nanoparticle Tracking Analysis (NTA) and TEM

**Figure 4:**
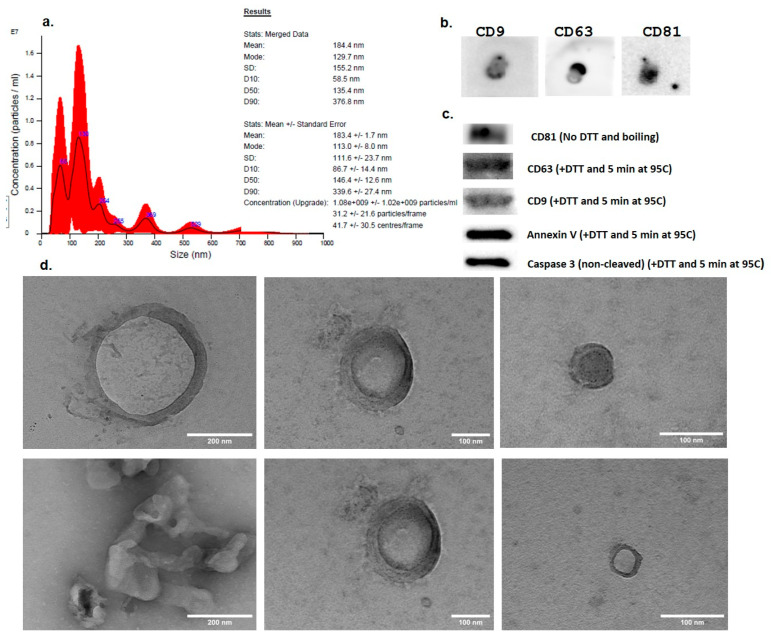
(**a**). Nanoparticle tracking analysis (NTA) of extracellular vesicles isolated from BM-MSC culture media. (**b**). Expression of EV markers CD9, CD63, and CD81 by Dot Blot immunoblotting and (**c**). positivity for the typical EV markers CD9, CD63, and CD81 but also for Annexin V and caspase 3, as shown on Western Blot images. (**d**). High resolution transmission electron microscopy (TEM) images of EVs isolated from BM-MSC culture medium. (N = 3).

The EV suspension was analyzed by NTA. The measurements showed that the isolated EVs had mean dimensions of 184.4 ± 1.7 nm ranging from 58.5 nm to 339.6 nm. The concentration of the EV suspension obtained from the culture medium of BM-MSCs was 180 × 10^6^ ± 102 × 10^6^ particles/mL (Figure 4a).

The TEM analysis of the EV samples showed the presence of intact vesicular structures with lipid-bilayer membranes having similar sizes to those measured by NTA, ranging from 50 to 250 nm (Figure 4d).

The protein concentration in the EV suspension in PBS (4 mL) obtained from 90 mL medium (180 × 10^6^ cells) as evaluated by BCA assay was 0.456 µg/μL.

#### 3.3.2. Expression of EV Markers—Dot Blot and WB

After the samples were passively loaded to the membrane and stained with the reversible stain Ponceau Red, the protein blots were visually observed. After probing with the primary antibodies (anti-CD9, anti-CD63, and anti-CD81) and the secondary HRP-linked antibodies were added, the images of the obtained protein blots showed positivity for the three tetraspanins (Figure 4b).

In the WB assay, following the migration of the samples, transfer on PVDF membranes, blocking and staining with the specific antibodies, the images recorded on C300 Azure Biosystem chemidoc showed intense bands in the specific regions for the three tetraspanins. CD63 and CD9 showed the most intense bands when using 50 mM dithiotreitol (DTT) loading buffer at 95 °C, whereas the best conditions for CD81 were with the loading buffer without DTT (Figure 4c). We obtained intense bands for Annexin V and caspase 3, which are considered specific markers for apoptotic extracellular vesicles, but no HSP70 band was obtained.

### 3.4. Assessment of the Protective Effect of EVs on HEI-OC1 Cells

#### 3.4.1. Internalization of PKH-Labeled EVs in HEI-OC1 Cells—Fluorescence Microscopy

**Figure 5:**
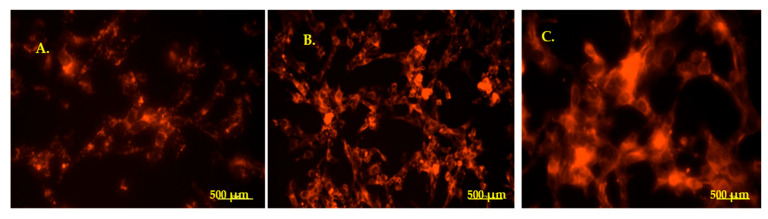
HEI-OC1 cells containing PKH26-labeled EVs. (**A**). 50 µL. (**B**). 100 µL. (**C**). 150 µL. Magnification 40×. Scale bars: 500 µm.

The PKH26-labeled EVs were observed in the HEI-OC1 cells after 24 h incubation, the internalized amount of fluorescent EVs (fluorescence intensity) increasing with the concentration (Figure 5).

#### 3.4.2. Assessment of ROS Generation

**Figure 6:**
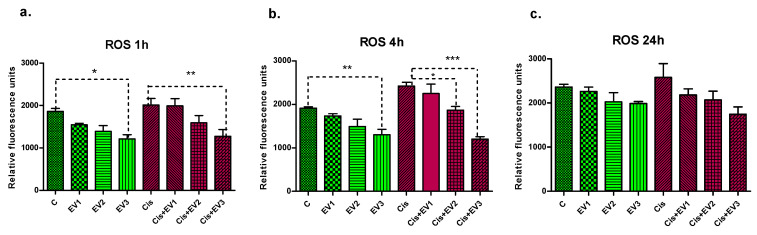
Concentration-dependent reduction of intracellular ROS produced by Cisplatin in HEI-OC1 cells measured at different time-points: (**a**). After 1 h, EV3 reduced intracellular ROS significantly compared to control cells (*p* < 0.05, one-way ANOVA, Bonferroni post-test) and highly significantly in Cisplatin-treated cells *p* < 0.001. (**b**). After 4 h incubation, EV3 reduced ROS in HEI-OC1 cells highly significantly (*p* < 0.001); in Cisplatin-treated cells, there was a significant reduction of ROS by EV2 (*p* < 0.05) and highly significant reduction of ROS by EV3 (*p* < 0.0001). (**c**). There was a 24 h reduction of ROS by all concentrations of EVs, but the effect is not statistically significant. * *p* < 0.05. ** *p* < 0.001. *** *p* < 0.0001.

The ROS generated by Cisplatin in the HEI-OC1 cells were assessed by the DCFDA assay at 1 h, 4 h, and 24 h incubation. At all readings, Cisplatin induced ROS in HEI-OC1 cells, while the pretreatment with EVs reduced the amount of produced ROS proportionally with the EV concentration (Figure 6). After 1 h incubation, all three concentrations of EVs reduced ROS compared to the non-treated cells, with EV3 reducing them significantly (*p* < 0.05, one-way ANOVA, Bonferroni post-test). Also in Cisplatin-treated cells, after 1 h incubation, the pretreatment with EV3 reduced ROS highly significantly (*p* < 0.001) (Figure 6a). At the 4 h reading, the different concentrations of EVs reduced ROS in HEI-OC1 cells compared to the control cells in a concentration-dependent manner, this effect being significant for EV3 (*p* < 0.001). (Figure 6b) EV2 and EV3 pretreatment before Cisplatin reduced ROS significantly compared those treated with Cisplatin only: 23% reduction for EV2 and 50.3% for EV3 (*p* < 0.001 for EV2 and *p* < 0.0001 for EV3, one-way ANOVA, Bonferroni multiple comparisons test). At 24 h, the dose-dependent differences, although present, were not significant (Figure 6c).

#### 3.4.3. Evaluation of the Protection of EVs Against Cisplatin Cytotoxicity—Alamar Blue

**Figure 7:**
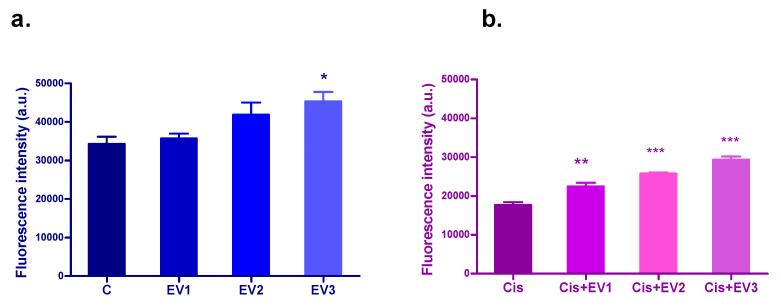
(**a**). The effect of different concentrations of the EV suspensions on HEI-OC1 cells’ viability after 24 h incubation, with the Alamar blue test: (**a**). increase in cell viability, statistically significant for EV3 (*p* < 0.05) (**b**). Dose-dependent protection conferred by the EV suspensions for the HEI-OC1 cells against Cisplatin toxicity, statistically significant: *p* < 0.001 for EV1 and *p* < 0.0001 for EV2 and EV3. * *p* < 0.05. ** *p* < 0.001. *** *p* < 0.0001.

The administration of increasing dilutions of EVs to the HEI-OC1 cell line after 24 h incubation increased viability in the treated cells compared to the control cells in a dose-dependent manner, the effect being significant for EV3 (*p* < 0.5, one-way ANOVA, Dunnett’s multiple comparison test) (Figure 7a). Cisplatin treatment significantly reduced the viability of HEI-OC1 cells after 24 h incubation. (*p* < 0.0001, one-way ANOVA, Bonferroni comparison post-test). All the concentrations of EVs administered to the HEI-OC1 cells as a pretreatment 30 min before Cisplatin increased viability dose-dependently, with highly significant differences compared to the cells treated only with Cisplatin. (*p* < 0.001 EV10, *p* < 0.0001 for EV20 and EV30) (Figure 7b).

#### 3.4.4. Evaluation of Apoptosis

**Figure 8:**
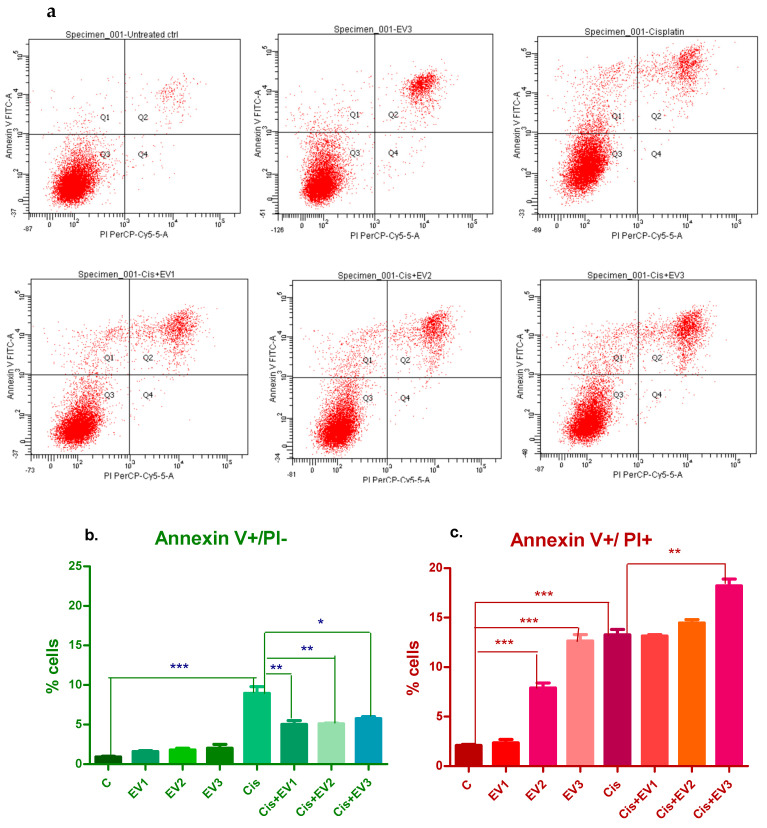
Apoptosis assessed after 24 h. (**a**). Flow cytometry plots showing the distribution of Annexin V (y axis)- and PI (x axis)-labeled cells after the treatment of HEI-OC1 cells with EVs, Cisplatin, and EVs followed by Cisplatin. Lower row: (**b**). Representation of the percentages of Annexin V-labeled cells (cells in early apoptosis) and of the differences between the applied treatments. Significant differences between control and Cisplatin-treated cells, (*p* < 0.0001 one-way ANOVA, Bonferroni post-test) and between Cisplatin alone and the combined treatments (EVs followed by Cisplatin—*p* < 0.001 for EV1 and EV2 and *p* < 0.05 for EV3); (**c**). Annexin V- and PI-labeled cells are significantly increased compared to control cells for all the treatments (except EV1 alone). The combined treatment (EVs before Cisplatin) led to an additional increase in the percentage of dead cells compared to Cisplatin alone, significant for EV3 (*p* < 0.001, one-way ANOVA, Bonferroni post-test). * *p* < 0.05. ** *p* < 0.001. *** *p* < 0.0001.

After 4 h incubation, there was no significant apoptosis induced by any treatment. After 24 h, the EV suspensions did not produce significant increases in the percentage of the cells in early apoptosis (positive for Annexin V). Cisplatin induced a highly significant increase in Annexin V positive cells (8.7%) (*p* < 0.0001, one-way ANOVA, Dunnett’s post-test). The pretreatment with EV suspensions reduced early apoptotic cells for all the concentrations, and the reduction was statistically significant (Figure 8a).

The percentage of cells labeled with both Annexin V and PI, which can be cells in late apoptosis or dead cells resulting from other mechanisms (necrosis), was significantly higher compared to the control for the treatments with EV2 (8.4%), EV3 (13.7%), and Cisplatin (12.7%). The pretreatment with EVs before Cisplatin resulted in significantly higher percentages of Annexin V+ and PI+ cells compared to the control cells, with EV1 and EV2 being similar to that of Cisplatin alone, while EV3 followed by Cisplatin led to a significant increase in late apoptotic cells (17.9%) compared to Cisplatin alone (Figure 8b).

## 4. Discussion

SNHL is an important public health issue, considering the important number of patients suffering from this debilitating condition. Hearing loss produced by exposure to ototoxic drugs (Cisplatin), extreme noise, autoimmune diseases, advanced age, etc., is a consequence of either the inner ear cells or acoustic neurons’ damage. As the available medical treatments are scarce and show poor efficiency, researchers seek new treatment methods, among which are biological therapies with MSCs and their derivatives, including extracellular vesicles [17].

Treatment in SNHL is challenging due to the reduced size of the inner ear, the anatomical difficulties of drugs reaching its structures, and the presence of the blood-labyrinth barrier. Another impediment is that the inner ear sensorineural cells have very limited regeneration capacity in adult mammals. The use of MSC-derived EVs, which proved their regenerative and immunomodulatory properties in several studies, could represent a solution for these problems.

In our study, the HEI-OC1 inner ear cell line was treated with BM-MSC-derived EVs, and their effects on HEI-OC1 viability, the production of ROS, and apoptosis as well as their potential to protect inner ear hair cells against Cisplatin toxicity were evaluated. The main mechanism of Cisplatin toxicity is the production of DNA damage represented by DNA adducts, but cell death occurs also as a result of oxidative stress, after the formation of ROS in the cochlea, which consecutively induce mitochondrial oxidative damage and activate apoptosis [18,19].

The EVs isolated in this study by the precipitation method had mean dimensions of 184.4 nm (in the range between 58.5 nm and 339.6 nm), as shown both by NTA and TEM, and showed positivity for CD9, CD63, and CD81 tetraspanins (WB) which are characteristic for extracellular vesicles. In the WB assay, intense bands were visible for Annexin V and caspase 3, which are considered specific markers for apoptotic EVs (apoEVs) but not for HSP70 which has an important role in maintaining protein homeostasis and the mediation of stress responses, apoptosis, and inflammation. EVs added to HEI-OC1 cells did not increase ROS production and slightly stimulated cellular viability. When EVs were added to HEI-OC1 cells 30 min before Cisplatin, they showed a protective effect by reducing ROS production and increasing cellular viability as demonstrated also in other studies using EVs isolated from MSCs to protect inner ear hair cells affected by ototoxic treatments in vitro [20,21] and in vivo [22] in the auditory hair cells. In a study by Park DJ et al. (2021), the protective effect of MSC-derived exosomes against Cisplatin toxicity is explained by the enrichment of HSP70 in the exosomes [15]. However, in our study, the WB assay did not show the expression of HSP70. To identify the proteins in MSC-derived apoEVs, Huang et al., 2024, used shotgun proteomics techniques, finding that apoEVs were enriched with oxidoreductase, taking part in the oxidative phosphorylation maintaining the redox state [23].

We obtained conflicting results when assessing apoptosis at 24 h: the cells in early apoptosis, when phosphatidylserine is exposed and the membranes are still intact, were not significantly increased by EVs. EV pretreatment before Cisplatin significantly reduced the Annexin V+ cells’ percentage. The fraction of cells in the later stage of apoptosis (cells with damaged membranes permitting PI entrance into the cells) increased after treatment with EVs alone proportionally with the EV concentration and even more when added before Cisplatin. This behavior contradicts the results obtained in other studies where EV protected against apoptosis [24]. The reason for this difference could be the different experimental conditions: EV isolation methods, the parent cells conditions, time-point for analysis, etc. In our study, the EVs were obtained from the serum-free cell culture medium harvested from MSCs. These cells were cultured serum free for 24 h. Serum starvation can induce apoptosis in some of the MSCs as demonstrated in a study in which serum deprivation induced apoptosis in a conjunctival epithelial cell line involving the caspase cascade [25]. The presence of caspase 3 in the EVs isolated in our study could explain the obtained results.

EVs excreted by healthy cells are heterogeneous in dimensions and origin and EVs produced by apoptotic cells must contain apoptotic extracellular vesicles (ApoEVs) among other types of EVs [26]. The EVs isolated from serum-starved MSCs, contained both EVs and ApoEVs with different cargos as well as different markers, and as a results had different effects on apoptosis, reducing the percentage of Annexin V positive cells (apoptotic) by probably the “healthy” EVs but enhancing the percentage of dead cells (late apoptosis, necrosis), probably the effect of the apoEVs.

ApoEVs are classified based on their dimensions in apoptotic bodies (ApoBD) with diameters between 1000 and 5000 nm and apoptotic microvesicles (ApoMVs) or exosome-like ApoEVs with dimensions between 50 and 1000 nm [27]. Several recent studies found that ApoEVs can promote tissue regeneration including skin regeneration and vascular protection [28,29] or wound healing [30].

Apoptosis is regulated by two mitochondrial pathways: caspase-dependent and caspase-independent. The caspase-dependent pathway is based on the activity of caspase 3, the Bcl family, and cytochrome c (Cyt c), while the caspase-independent pathway is PARP-1-mediated cell death, or “regulated necrosis” [31] One study found that ApoEVs (under 1000 nm) promoted stem cell proliferation, migration, differentiation, and wound healing in a diabetes mouse model, while apoptotic bodies (with diameter >1000 nm) had the opposite effect. Apoptosis is a complex process, proving to be more than a “silent” type of cell death but an active form of communication from dying cells to live cells EV [32]. Apoptosis is no longer considered a form of ‘silent’ cell death considering its active communication with the neighboring cells. It has an important contribution to tissue homeostasis by regulating survival and tissue remodeling [33,34]. How these signals are transmitted to the surrounding healthy cells remains largely unknown. Brock et al., 2019 [35], showed that the communication of dying cells with their neighbors is through apoptotic bodies containing Wnt8a. Wnt signaling is activated in the recipient cells, stimulating cell division [35,36].

In our study, the EV suspensions with origins in serum-deprived cells contained a fraction of ApoEVs which could be responsible for the induction of additional apoptosis in the Cisplatin-treated HEI-OC1 cells. The activation of apoptosis can be considered, in this case, as a protecting mechanism aiming to eliminate damaged cells, while, in the meantime, stimulating cell proliferation as demonstrated in the viability experiments.

Our study has several limitations as it describes the effects of EVs using only the in vitro model (HEI-OC1 cell line). It also lacks deeper insights into the mechanisms involved in the studied endpoints, comparisons between EVs from healthy cells and apoptotic cells as well as evaluation of the effects on other biological models such as ex vivo cultures of Corti organs and in vivo studies to confirm the findings.

## 5. Conclusions

Extracellular vesicles have a proved great potential in the cell-free therapy of several diseases, including SNHL. Our study showed that EVs secreted by MSCs were able to protect inner ear hair cells against Cisplatin toxicity by reducing ROS production, stimulating cell proliferation, and stimulating the elimination of damaged cells by apoptosis. The mechanisms involved in the process of EVs/ApoEVs regulating apoptosis, proliferation, and survival of cells need to be further explored by more comprehensive research at molecular level in order to permit safe EV treatments at their full potential. In addition, the effects of EVs should also be investigated in animal models.

## Data Availability

Data is contained within the article. Further inquiries can be directed to the corresponding author(s).

## Abbreviations

The following abbreviations are used in this manuscript:

BM-MSCs	Bone-marrow mesenchymal stem cells
EVs	Extracellular vesicles
apoEVs	Apoptotic extracellular vesicles
SNHL	Sensorineural hearing loss

## References

[1] Deafness and Hearing Loss, World Health Organization. https://www.who.int/news-room/fact-sheets/detail/deafness-and-hearing-loss.

[2] Sarow A, Important Hearing Loss Statistics and Studies 2023, Soundly. https://www.soundly.com/blog/hearing-loss-statistics.

[3] Hopkins K., Michael J., Aminoff F.B., Dick F.S. (2015). Handbook of Clinical Neurology Chapter 27—Deafness in Cochlear Auditory Nerve Disorders.

[4] Liu F., Han B., Zhou X., Huang S., Huang J. (2023). Research progress on the treatment and nursing of sensorineural hearing loss. Front. Neurosci..

[5] Lieu J.E.C., Kenna M., Anne S., Davidson L. (2020). Hearing loss in children: A review. JAMA.

[6] Fang Q., Wei Y., Zhang Y., Cao W., Yan L., Kong M., Zhu Y., Xu Y., Guo L., Zhang L. (2023). Stem cells as potential therapeutics for hearing loss. Front. Neurosci..

[7] Doyle L.M., Wang M.Z. (2019). Overview of Extracellular Vesicles, Their Origin, Composition, Purpose, and Methods for Exosome Isolation and Analysis. Cells.

[8] Sheta M., Taha E.A., Lu Y., Eguchi T. (2023). Extracellular Vesicles: New Classification and Tumor Immunosuppression. Biology.

[9] Kim H.I., Park J., Zhu Y., Wang X., Han Y., Zhang D. (2024). Recent advances in extracellular vesicles for therapeutic cargo delivery. Exp. Mol. Med..

[10] Jeon S.J., Oshima K., Heller S., Edge A.S. (2007). Bone marrow mesenchymal stem cells are progenitors in vitro for inner ear hair cells. Mol. Cell Neurosci..

[11] Jang S., Cho H.H., Kim S.H., Lee K.H., Jun J.Y., Park J.S., Jeong H.S., Cho Y.B. (2015). Neural-Induced Human Mesenchymal Stem Cells Promote Cochlear Cell Regeneration in Deaf Guinea Pigs. Clin. Exp. Otorhinolaryngol..

[12] Ma Y., Guo W., Yi H., Ren L., Zhao L., Zhang Y., Yuan S., Liu R., Xu L., Cong T. (2016). Transplantation of human umbilical cord mesenchymal stem cells in cochlea to repair sensorineural hearing. Am. J. Transl. Res..

[13] Kada S., Hamaguchi K., Ito J., Omori K., Nakagawa T. (2020). Bone Marrow Stromal Cells Accelerate Hearing Recovery via Regeneration or Maintenance of Cochlear Fibrocytes in Mouse Spiral Ligaments. Anat. Rec..

[14] Scheper V., Hoffmann A., Gepp M.M., Schulz A., Hamm A., Pannier C., Hubka P., Lenarz T., Schwieger J. (2019). Stem Cell Based Drug Delivery for Protection of Auditory Neurons in a Guinea Pig Model of Cochlear Implantation. Front. Cell. Neurosci..

[15] Park D.J., Park J.E., Lee S.H., Eliceiri B.P., Choi J.S., Seo Y.J. (2021). Protective effect of MSC-derived exosomes against cisplatin-induced apoptosis via heat shock protein 70 in auditory explant model. Nanomedicine.

[16] Gopalarethinam J., P Nair A., Iyer M., Vellingiri B., Subramaniam M.D. (2023). Advantages of mesenchymal stem cell over the other stem cells. Acta Histochem..

[17] Perde-Schrepler M., Ioana B. Mesenchymal Stem Cell- Derived Exosomes as Cell-Free Therapeutics for Sensorineural Hearing Loss. Biomol Biomed [Internet]. 2025 Mar. 6 [cited 2025 Apr. 30]. https://www.bjbms.org/ojs/index.php/bjbms/article/view/11517.

[18] Perde-Schrepler M., Fischer-Fodor E., Virag P., Brie I., Cenariu M., Pop C., Valcan A., Gurzau E., Maniu A. (2020). The expression of copper transporters associated with the ototoxicity induced by platinum-based chemotherapeutic agents. Hear. Res..

[19] Zhang L., Du Z., He L., Liang W., Liu K., Gong S. (2022). ROS-Induced Oxidative Damage and Mitochondrial Dysfunction Mediated by Inhibition of SIRT3 in Cultured Cochlear Cells. Neural Plast..

[20] Lai R., Cai C., Wu W., Hu P., Wang Q. (2020). Exosomes derived from mouse inner ear stem cells attenuate gentamicin-induced ototoxicity in vitro through the miR-182-5p/FOXO3 axis. J. Tissue Eng. Regen. Med..

[21] Liu H., Kuang H., Wang Y., Bao L., Cao W., Yu L., Qi M., Wang R., Yang X., Ye Q. (2024). MSC-derived exosomes protect auditory hair cells from neomycin-induced damage via autophagy regulation. Biol. Res..

[22] Park S.J., Kim J.M., Kim J., Hur J., Park S., Kim K., Shin H.J., Chwae Y.J. (2018). Molecular mechanisms of biogenesis of apoptotic exosome-like vesicles and their roles as damage-associated molecular patterns. Proc. Natl. Acad. Sci. USA.

[23] Huang X., Kou X., Zhan T., Wei G., He F., Mao X., Yang H. (2023). Apoptotic vesicles resist oxidative damage in noise-induced hearing loss through activation of FOXO3a-SOD2 pathway. Stem Cell Res. Ther..

[24] Higuchi A., Shimmura S., Takeuchi T., Suematsu M., Tsubota K. (2006). Elucidation of apoptosis induced by serum deprivation in cultured conjunctival epithelial cells. Br. J. Ophthalmol..

[25] Gregory C.D., Rimmer M.P. (2023). Extracellular vesicles arising from apoptosis: Forms, functions, and applications. J. Pathol..

[26] Atkin-Smith G.K., Tixeira R., Paone S., Mathivanan S., Collins C., Liem M., Goodall K.J., Ravichandran K.S., Hulett M.D., Poon I.K. (2015). A novel mechanism of generating extracellular vesicles during apoptosis via a beads-on-a-string membrane structure. Nat. Commun..

[27] Chen H., Kasagi S., Chia C., Zhang D., Tu E., Wu R., Zanvit P., Goldberg N., Jin W. (2019). Extracellular vesicles from apoptotic cells promote TGFbeta production in macrophages and suppress experimental colitis. Sci. Rep..

[28] Liu H., Liu S., Qiu X., Yang X., Bao L., Pu F., Liu X., Li C., Xuan K., Zhou J. (2020). Donor MSCs release apoptotic bodies to improve myocardial infarction via autophagy regulation in recipient cells. Autophagy.

[29] Liu J., Qiu X., Lv Y., Zheng C., Dong Y., Dou G. (2020). Apoptotic bodies derived from mesenchymal stem cells promote cutaneous wound healing via regulating the functions of macrophages. Stem Cell Res. Ther..

[30] Zhang X., Yang J., Ma S., Gao X., Wang G., Sun Y., Yu Y., Wang Z., Tian W., Liao L. (2024). Functional diversity of apoptotic vesicle subpopulations from bone marrow mesenchymal stem cells in tissue regeneration. J. Extracell. Ves..

[31] Lee C.K., Shin J.I., Cho Y.S. (2011). Protective effect of minocycline against cisplatin-induced ototoxicity. Clin. Exp. Otorhinolaryngol..

[32] Kakarla R., Hur J., Kim Y.J., Kim J. (2020). Apoptotic cell-derived exosomes: Messages from dying cells. Exp. Mol. Med..

[33] Perez-Garijo A., Steller H. (2015). Spreading the word: Non-autonomous effects of apoptosis during development, regeneration and disease. Development.

[34] Fu Y., Sui B., Xiang L., Yan X., Wu D., Shi S., Hu X. (2021). Emerging understanding of apoptosis in mediating mesenchymal stem cell therapy. Cell Death Dis..

[35] Brock C.K., Wallin S.T., Ruiz O.E., Samms K.M., Mandal A., Sumner E.A., Eisenhoffe G.T. (2019). Stem cell proliferation is induced by apoptotic bodies from dying cells during epithelial tissue maintenance. Nat. Commun..

[36] Li M., Liao L., Tian W. (2020). Extracellular Vesicles Derived from Apoptotic Cells: An Essential Link Between Death and Regeneration. Front. Cell Dev. Biol..

